# Repeated Nitrogen Dioxide Exposures and Eosinophilic Airway Inflammation in Asthmatics: A Randomized Crossover Study

**DOI:** 10.1289/ehp.1307240

**Published:** 2014-04-18

**Authors:** Véronique Ezratty, Gaëlle Guillossou, Catherine Neukirch, Monique Dehoux, Serge Koscielny, Marcel Bonay, Pierre-André Cabanes, Jonathan M. Samet, Patrick Mure, Luc Ropert, Sandra Tokarek, Jacques Lambrozo, Michel Aubier

**Affiliations:** 1 Service des Etudes Médicales (SEM), EDF, Levallois-Perret, France; 2 Service de Pneumologie A, Clinical Center of Investigation, Inserm U700, Bichat Hospital, Université Paris Diderot, DHU FIRE, Paris, France; 3 Laboratoire de Biochimie, Bichat Hospital, Paris, France; 4 Service de biostatistiques et épidémiologie, Gustave Roussy, Villejuif, France; 5 Department of Physiology, Bichat Hospital, Paris, France; 6 Department of Preventive Medicine, Keck School of Medicine of the University of Southern California, Los Angeles, Los Angeles, California, USA; 7 Center for Research & Innovation in Gas and New Energy Sources, CRIGEN, GDF SUEZ, St-Denis, France

## Abstract

Background: Nitrogen dioxide (NO_2_), a ubiquitous atmospheric pollutant, may enhance the asthmatic response to allergens through eosinophilic activation in the airways. However, the effect of NO_2_ on inflammation without allergen exposure is poorly studied.

Objectives: We investigated whether repeated peaks of NO_2_, at various realistic concentrations, induce changes in airway inflammation in asthmatics.

Methods: Nineteen nonsmokers with asthma were exposed at rest in a double-blind, crossover study, in randomized order, to 200 ppb NO_2_, 600 ppb NO_2_, or clean air once for 30 min on day 1 and twice for 30 min on day 2. The three series of exposures were separated by 2 weeks. The inflammatory response in sputum was measured 6 hr (day 1), 32 hr (day 2), and 48 hr (day 3) after the first exposure, and compared with baseline values measured twice 10–30 days before the first exposure.

Results: Compared with baseline measurements, the percentage of eosinophils in sputum increased by 57% after exposure to 600 ppb NO_2_ (*p* = 0.003) but did not change significantly after exposure to 200 ppb. The slope of the association between the percentage of eosinophils and NO_2_ exposure level was significant (*p* = 0.04). Eosinophil cationic protein in sputum was highly correlated with eosinophil count and increased significantly after exposure to 600 ppb NO_2_ (*p* = 0.001). Lung function, which was assessed daily, was not affected by NO_2_ exposure.

Conclusions: We observed that repeated peak exposures of NO_2_ performed without allergen exposure were associated with airway eosinophilic inflammation in asthmatics in a dose-related manner.

Citation: Ezratty V, Guillossou G, Neukirch C, Dehoux M, Koscielny S, Bonay M, Cabanes PA, Samet JM, Mure P, Ropert L, Tokarek S, Lambrozo J, Aubier M. 2014. Repeated nitrogen dioxide exposures and eosinophilic airway inflammation in asthmatics: a randomized crossover study. Environ Health Perspect 122:850–855; http://dx.doi.org/10.1289/ehp.1307240

## Introduction

Nitrogen dioxide (NO_2_), a ubiquitous atmospheric pollutant, is a respiratory irritant that remains a matter of concern [World Health Organization (WHO) European Centre for Environment and Health and WHO Regional Office for Europe 2013]. Indoor concentrations of NO_2_ often exceed those found outdoors, especially when unvented combustion appliances are used. Inside homes, peak levels of NO_2_, associated with the use of gas and solid-fuel appliances for cooking and heating, have been measured in the range of 80–1,100 ppb (150–2,090 μg/m^3^) (Basu and Samet 1999; Dennekamp et al. 2001; Kotzias et al. 2005; Pilotto et al. 1997). Outdoors, hourly NO_2_ concentrations in cities rarely exceed 200 ppb (380 μg/m^3^) (U.S. Environmental Protection Agency 2008), although urban levels can reach levels as high as 500 ppb (950 μg/m^3^) (WHO 2006), especially for short periods in streets with heavy traffic and in road tunnels (Larsson et al. 2010).

Epidemiological and controlled human exposure studies suggest that people with asthma are more susceptible to the effects of NO_2_ when compared with healthy individuals (Bauer et al. 1986; Belanger et al. 2006; Bylin et al. 1988; Hasselblad et al. 1992; Jorres and Magnussen 1990; Strand et al. 1996).

However, despite the extensive literature on NO_2_-induced health effects, some inconsistencies in the results of studies have been noted (Jarvis et al. 2010). In asthmatics, NO_2_ exposure without allergen challenge did not result in lung functional changes in most studies (Avol et al. 1988; Kleinman et al. 1983; Linn et al. 1986; Mohsenin 1987), and inconsistent results were found in airway responsiveness after nonspecific bronchoconstrictor challenges (Bylin et al. 1988; Hazucha et al. 1983; Jorres and Magnussen 1991; Kleinman et al. 1983; Roger et al. 1990; Strand et al. 1996). After allergen challenge, exposure to NO_2_ in asthmatics increased airway hyperresponsiveness (Jenkins et al. 1999; Strand et al. 1997, 1998; Tunnicliffe et al. 1994) and eosinophilic inflammation (Barck et al. 2002, 2005).

A few studies have investigated the inflammatory response to a single exposure of NO_2_ without allergen challenge in asthmatics, but the findings have been inconsistent (Jorres et al. 1995; Solomon et al. 2004; Vagaggini et al. 1996).

We investigated whether repeated brief exposures to 200 ppb (380 μg/m^3^) and 600 ppb (1,130 μg/m^3^) NO_2_, which mimic indoor NO_2_ peaks, enhance airway inflammation in asthmatics. This clinical study involved 19 adults with intermittent asthma and used a randomized double-blind protocol with assessment of inflammatory response in induced sputum.

## Materials and Methods

*Participants*. Nineteen patients [14 men and 5 women; median age, 29 years; age range, 20–69 years; median body mass index (BMI), 26 kg/m^2^; BMI range, 20–39 kg/m^2^] were included in the study (Table 1). All had intermittent asthma as defined by the Global Initiative for Asthma (2010) guidelines and were nonsmokers (18 had never smoked and 1 had stopped smoking some 10 years earlier). Only patients who had a diagnosis of asthma confirmed by a positive methacholine challenge performed twice at baseline 10–30 days before the first exposure were included in the study. A positive methacholine test was defined as a methacholine provocative dose causing a 20% decrease in forced expiratory volume in 1 sec (FEV_1_) from control FEV_1_ (PD_20_ methacholine) < 4 mg. All participants had allergy to house dust mites (HDM) and/or pollen confirmed by a positive skin-prick test done ≥ 4 weeks before their inclusion in the study. The study was performed outside the pollen season for those who had been diagnosed with pollen allergies. Of the 19 participants, 6 had a personal history of atopic dermatitis and/or an atopic familial history. None of those six used inhaled or oral corticosteroids or other antiinflammatory therapy, and the only permitted medication was an inhaled β_2_-agonist, used as needed during the study period (from baseline, i.e., 30 days before the first NO_2_ exposure until the end of the study 2 weeks after the last exposure). Participants who had gas stoves and/or unvented combustion appliances at home were told not to use them at baseline and on the days of exposures and during the 2 days before and after NO_2_ exposure. All participants had to be free of airway infection for at least 6 weeks before baseline measurements.

**Table 1:** Characteristics of participants.

Participant	Age (years)	History of atopy^*a*^	Smoking status	Sex	Height (cm)	Weight (kg)	BMI (kg/m^2^)	Asthma duration (years)	FEV_1_ at inclusion (FEV_1_ % predicted)	PD20 methacholine at baseline (μg)	Percent eosinophils in sputum at baseline^*b*^
1^*c*^	26	Yes	N	F	164	57	21	8	3.23 (110)	1,600	NA
2	29	No	E	M	185	90	27	11	4.13 (89)	1,490	14.73
3	29	No	N	M	182	87	27	10	4.43 (99)	690	11.88
4	31	No	N	M	161	84	33	8	2.87 (81)	3,200	6.79
5	28	No	N	M	174	74	25	4	3.69 (88)	1,550	1.68
6	27	No	N	M	180	88	28	21	3.57 (80)	1,220	0.93
7	24	No	N	M	178	70	22	8	3.88 (87)	1,070	4.25
8	30	No	N	F	159	56	22	20	3.03 (103)	500	2.68
9	29	Yes	N	M	168	72	26	22	3.89 (100)	3,200	1.88
10^*c*^	28	Yes	N	M	186	92	27	2	4.49 (96)	800	ANR
11	20	Yes	N	F	158	49	20	9	2.29 (76)	930	0.26
12	69	No	N	M	178	90	29	5	2.49 (78)	3,200	17.72
13	30	No	N	F	163	55	21	20	2.68 (87)	1,950	32.08
14	32	Yes	N	M	179	82	26	24	4.12 (92)	340	2.51
15	24	No	N	F	171	60	21	14	3.41 (97)	310	5.12
16	28	No	N	M	174	62	21	16	3.82 (91)	2,100	20.58
17	32	No	N	M	176	115	38	21	4.16 (100)	2,170	2.99
18^*c*^	30	Yes	N	M	169	89	32	21	3.75 (95)	190	NA
19	30	No	N	M	174	115	39	23	3.16 (76)	210	1.55
Abbreviations: ANR: available but not relevant because participant 10 did not complete the three series of exposure; E, ex-smoker; N, never-smoker; NA: not available. ^***a***^Personal history of atopic dermatitis and/or atopic familial history. ^***b***^Percent eosinophils at baseline (10–30 days before first exposure). ^***c***^Participants excluded from the analysis: participants 1 and 18 did not produce adequate sputum specimens for cell analysis at baseline (squamous cells > 20%); participant 10 did not complete the three series of exposure.											

The study was registered by the French Ministry of Health (DGS 2006/0016) and approved by the Ethics Committee of Hotel-Dieu Hospital, Paris, France (project 0611398, registered on 28 February 2007). All participants signed informed consent forms before enrollment in the study.

**Figure 1:**
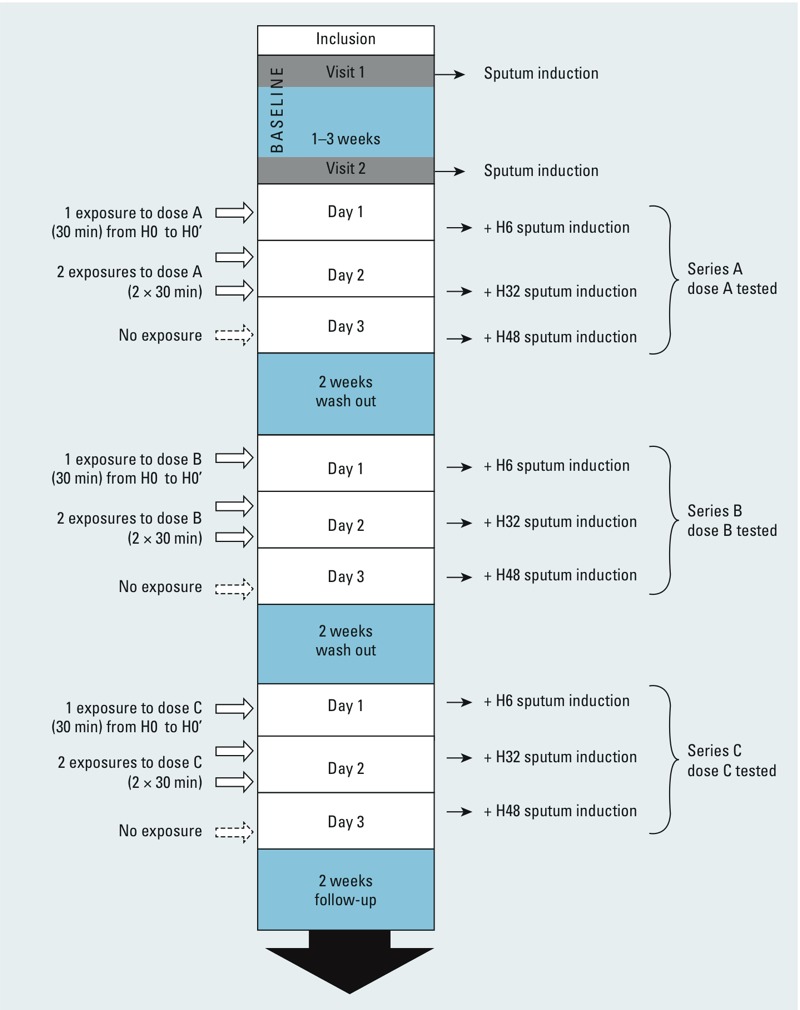
Flow diagram of the study period for each participant. Abbreviations: H0, immediately before 1st exposure (day 1); H0’, immediately after 1st exposure (day 1); H6, 6 hr after H0’ (day 1); H32, 32 hr after H0’ (day 2); H48, 48 hr after H0’ (day 3). The dose order was attributed randomly; so, series A, B, and C ­corresponds to clean air, 200-ppb NO_2_, or 600-ppb NO_2_ exposure.

**Figure 2:**
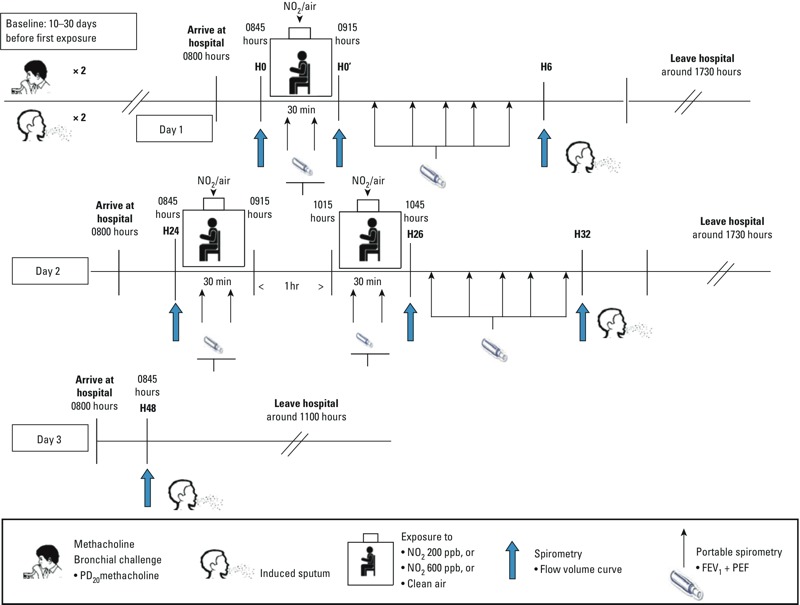
Study design for each of the three series of exposures separated by 2 weeks. Abbreviations: H0, immediately before 1st exposure (day 1); H0’, immediately after 1st exposure (day 1); H6, 6 hr after H0’ (day 1); H32, 32 hr after H0’ (day 2); H48, 48 hr after H0’ (day 3). Participants were exposed at rest in a double-blinded, randomized, crossover design to clean air, 200-ppb NO_2_, or 600 ppb NO_2_ once for 30 min on day 1, and twice for 30 min on day 2. There was no exposure on day 3. Time of day is expressed in military form.

*Study design*. The study had a double-blind, crossover design, with each participant acting as his or her own control. For each participant, the study involved three series each of three exposures at rest: one series to clean air, one series to 200 ppb (380 μg/m^3^) NO_2_, and one series to 600 ppb (1,130 μg/m^3^) NO_2_. The order of the three series of exposures was randomized (Figure 1). The design for each series of exposures with the timing of pulmonary function testing, and sputum inductions from day 1 to day 3 is described in Figure 2. For each series, participants were exposed to the same level of NO_2_ or to clean air once for 30 min on day 1, and twice for 30 min on day 2, at the same time and on the same days of the week. The two exposures performed on day 2 were separated by 1 hr. There was no exposure on day 3. There was an interval of 2 weeks between each series of exposures. Only the engineer in charge of injection into the chamber knew whether the participant was being exposed to NO_2_ or clean air.

Sputum was induced twice at baseline (10–30 days before first exposure, with 1–3 weeks between the sputum inductions) and for each series of exposures, 6 hr (on day 1), 32 hr (on day 2), and 48 hr (on day 3) after the end of the first exposure. Spirometry with flow–volume curves was carried out at baseline (immediately before first exposure) and daily from day 1 to day 3, before and immediately after exposures, and immediately before sputum inductions. Allergen challenge was not performed, either before or after the exposures.

*Nitrogen dioxide/clean air exposure*. The exposures were performed in an 8.8-m^3^ exposure chamber installed in the investigation clinical center at the Hospital Bichat in Paris as previously described (Ezratty et al. 2007). The chamber was supplied with fresh, particle-free air at a mean temperature of 25°C and a mean relative humidity of 32%. The air supply passed through HEPA and activated carbon filters. We used NO_2_ concentrated at 950 ppm compressed in a 20-L gas bottle under 150 bar pressure for the 600-ppb exposures, and a gas bottle of NO_2_ concentrated at 520 ppm under 150 bar pressure for the 200-ppb exposures (Air Liquide SA, Paris, France). A mass flow meter secured the stability of the injected flow at the expected concentration (200 ppb or 600 ppb).

During exposures, NO_2_ concentration inside the exposure chamber was continuously monitored (chemiluminescence oxides of nitrogen analyzer, Model AC 32 M; Environnement SA, 78300 Poissy, France). The mean concentration was 581 ppb ± 3.2% for 600-ppb NO_2_ exposures and 203 ppb ± 1.5% for 200-ppb NO_2_ exposures. During exposures to clean air, the NO_2_ concentration was < 10 ppb.

*Pulmonary function and methacholine challenge testing*. Flow-volume curves were obtained using a Biomedin spirometer (Biomedin srl, Padova, Italy) according to the European Community Respiratory Health Survey specifications to determine FEV_1_ and peak expiratory flow (PEF) (Quanjer 1993).

Methacholine challenge tests were done twice at baseline with an automatic inhalation-synchronized Mefar MB3 dosimeter jet nebulizer (Mefar spa, Bovezzo, Italy), as described elsewhere (Aubier et al. 1992; Ezratty et al. 2007). Methacholine challenge tests at baseline were conducted 10–30 days before the start of the exposures to avoid any putative interference of the methacholine challenge with the effect of NO_2_ (Jorres et al. 1995).

*Sputum induction and inflammatory marker measurements.* We performed sputum induction with an aerosol of hypertonic saline using the method of Pin et al. (1992). The sputum was analyzed within 1 hr according to Pizzichini et al. (1996), as described elsewhere (Ezratty et al. 2007). Total nonsquamous inflammatory cell counts were expressed as 10^3^ cells/mg of induced sputum. Differential cell counts were performed by counting 400 cells on May Grünwald Giemsa stained slides by two expert observers blinded to the participant’s exposure. Results were expressed both as percentage and as number of inflammatory cells per milligram of induced sputum. Only samples with cell viability > 70% and squamous cell contamination < 20% were considered adequate.

Sputum supernatant concentrations of eosinophil cationic protein (ECP) levels were measured by a commercially available enzyme assay (CAP-FEIA; Pharmacia, St Quentin-en-Yvelines, France), with a lower detection limit of 2 ng/mL.

*Follow-up during the study period*. After 0, 15, and 30 min of exposure to NO_2_ or clean air in the chamber, participants were asked questions relating to respiratory symptoms and perception of discomfort.

FEV_1_ and PEF were monitored twice during exposure at 15-min intervals and hourly for 6 hr after leaving the chamber, with a portable combined spirometer (One Flow Tester; Mediflux, Croissy Beaubourg, France).

During the 10–30 days between baseline measurements and first exposure and during the 2-week interval following each series of exposures, subjective symptoms and medications were recorded every day. Each participant measured FEV_1_ and PEF twice daily with a portable combined spirometer.

*Sample size*. The primary end point was the change in the percentage of eosinophils in sputum, expressed as the ratio between the percentage after exposure and the baseline percentage. When the study was designed, literature reports were insufficient to determine the variance of the ratio, which was mandatory to estimate the sample size. Variance was estimated after inclusion of the first eight participants without unblinding. Based on the variance found of 0.10, a sample size of 18 participants was considered sufficient to demonstrate a doubling of the percentage of eosinophils in sputum with a power of 80% and a significance level of 0.05 (see Supplemental Material, Table S1). In addition, a doubling has been reported to be consistent with clinically relevant changes in clinical status of asthmatics (Green et al. 2002).

*Statistical analysis*. The parameters studied in sputum were the percentage of eosinophils, the number of eosinophils per milligram, ECP, the number of neutrophils per milligram, and the number of macrophages per milligram. All parameters were log-transformed to normalize the distributions. We used a generalized linear model (GLM procedure, SAS v9.4; SAS Institute Inc., Cary, NC, USA) for the analyses. We included in the statistical model the effect of the participant, the time, the dose, and the interaction between the time and the dose. The interpretation of the results was based on the type III sum of squares. In case of a significant interaction between day and dose, a global analysis was performed, followed by per-day analyses. In the global analysis, all the data concerning the parameter were summarized by their geometric means (GMs) (in case of sputum inductions, this relates to three sputum inductions over 3 days, one per day), and we tested the relation between NO_2_ concentration—used as a quantitative value (0, 200, and 600 ppb)—and the parameter studied. The global analysis provided the *p*_trend_. To display the results in a meaningful way, we also analyzed the data using classes of exposure (without any preconceived idea on the form of the relationship): For each class of exposure, we estimated the least-square mean (LSMEAN) and its 95% confidence limits. The LSMEANs and the confidence limits were exponentiated to obtain the changes relative to the baseline measurements and their confidence intervals (CIs). The per-day analyses were reported only when the trend in the global analysis was significant. *p*-Values < 0.05 were considered significant.

## Results

Among the 19 participants, 18 completed the three series of exposures and were included in the analysis. Among those, two did not produce an adequate sample of sputum at all time points and were not included in analyses of sputum (Table 1).

Respiratory function—FEV_1_ and PEF—measured by spirometry, did not significantly change after NO_2_ exposure compared with clean air exposure (Table 2). No major clinical adverse reactions, such as coughing, wheezing, or chest tightness suggesting asthma attacks, were observed during exposures or follow-up. During the 2 weeks after each exposure, subjective symptoms and peak flow measurements were not significantly different regardless of exposure.

**Table 2:** GMs (95% CIs) of values relative to baseline of FEV1 and PEF evaluated in the 18 participants who completed the study.

Variable	0 ppb NO_2_ (clean air)	200 ppb NO_2_	600 ppb NO_2_	*p*-Value
FEV_1_/baseline	1.02 (1.00, 1.05)	1.00 (0.97, 1.03)	1.00 (0.98, 1.03)	0.41
PEF/baseline	1.05 (0.99, 1.11)	1.04 (0.98, 1.10)	1.01 (0.96, 1.07)	0.36
Baseline values were measured immediately before first exposure (H0 on day 1) for each series of exposure. Each value corresponds to the GM change from baseline of the six values [H0’, immediately after first exposure (day 1); H6, 6 hr after H0’ (day 1); H32, 32 hr after H0’ (day 2); H48, 48 hr after H0’ (day 3)] obtained by spirometry with flow-volume curves. The effects of exposure, regardless of the dose, on FEV_1_ and PEF were not significant.				

The primary analysis tested the exposure level (dose), the time (because the study design included assessment at three time points after exposure, one each day) and the interaction between time and exposure level. The dose was significantly related to the change in the percentage of eosinophils, the time was not. There was a significant interaction between time and exposure level, meaning that the association between dose and effect on eosinophils was different according to the day. As planned in the case of a significant interaction, we performed both a global analysis, averaging the measurements over the 3 days, and per-day analyses.

In the global analysis, the slope of the association between the percentage of eosinophils and NO_2_ exposure level was significant (*p* = 0.04) (Table 3).

**Table 3:** Changes relative to baseline measurements (performed 10–30 days before the first exposure) for parameters measured in sputum [GM % (95% CI), *n* = 16].

Variable	0 ppb NO_2_ (clean air)	200 ppb NO_2_	600 ppb NO_2_	*p*_Trend_
Percentage of eosinophils	–12 (–34, 16)	–5 (–28, 26)	57 (18, 109)**	0.04
Day 1	16 (–28, 86)	–34 (–59, 6)	12 (–31, 79)	0.81
Day 2	–5 (–37, 44)	–3 (–36, 46)	102 (32, 211)**	0.01
Day 3	–39 (–67, 13)	36 (–26, 149)	79 (–3, 230)	0.03
No. of eosinophils/mg	–5 (–31, 29)	23 (–10, 68)	120 (60, 202)^#^	0.02
Day 1	–5 (–44, 60)	–24 (–55, 29)	64 (–3, 177)	0.10
Day 2	9 (–39, 95)	23 (–31, 119)	142 (32, 344)**	0.06
Day 3	–18 (–57, 56)	99 (5, 278)*	163 (38, 398)**	0.03
ECP	21 (–1, 47)	0 (–18, 22)	43 (17, 75)**	0.23
No. of neutrophils/mg	12 (–17, 51)	–9 (–32, 22)	10 (–19, 49)	0.97
No. of macrophages/mg	–18 (–33, 0)	–2 (–20, 21)	–6 (–24, 15)	0.46
GM percentages represent all changes of the parameter for the 3 days (one sputum induction per day). *p < 0.05; **p < 0.01; and ^#^*p* < 0.001, compared with baseline.				

Compared with baseline measurements, the percentage of eosinophils in sputum increased by 57% (95% CI: 18, 109%) after 600 ppb NO_2_ (*p* = 0.003) but did not change significantly after clean air or after 200 ppb NO_2_.

Similar results were found for the association between the number of eosinophils per milligram of sputum and NO_2_ exposure level. In the global analysis, the slope of the association was significant (*p* = 0.02) (Table 3). The number of eosinophils per milligram of sputum increased by 120% (95% CI: 60, 202%) after 600 ppb NO_2_ (*p* < 0.001) but not after clean air or after 200 ppb NO_2_ (Table 3).

Per-day analysis showed that the slope of the association between the percentage of eosinophils and NO_2_ exposure level was not significant at day 1 (*p* = 0.81), but was significant at day 2 (*p* = 0.01) and day 3 (*p* = 0.03). A similar pattern was found for the slope of the association between the number of eosinophils per milligram of sputum and the level of exposure to NO_2_, which was not significant at day 1 (*p* = 0.10), close to significance at day 2 (*p* = 0.06), and significant at day 3 (*p* = 0.03) (Table 3; see also Supplemental Material, Figure S1).

Compared with baseline measurements, there was no significant change of the percentage of eosinophils in sputum regardless of the level of exposure to NO_2_ at day 1. At day 2, the percentage and the number of eosinophils increased significantly at 600 ppb, but not at 200 ppb NO_2_. At day 3, the number of eosinophils increased significantly at 200 ppb and at 600 ppb NO_2_, and the increase of the percentage of eosinophils was close to significance at 600 ppb NO_2_, but not significant at 200 ppb (Table 3; see also Supplemental Material, Figure S1).

Absolute values not baseline adjusted [GMs (95% CIs)] of the percentages of eosinophils are reported in Supplemental Material, Table S2. Individual plots of the percentage of eosinophils are displayed in Supplemental Material, Figure S2. In order to see if results were driven by a participant in particular, we did a sensitivity analysis: a series of analyses with one different participant removed for each analysis. The trend test that measures the relationship between the dose of NO_2_ and the increase of the percentage of eosinophils in sputum from baseline was significant (*p* < 0.05) in 10 of the tests, and was close to significance (*p* < 0.10) for the other participants. Moreover, the increase of the percentage of eosinophils in sputum compared with baseline measurements was always significant at 600 ppb, regardless of which participant was removed from the analysis (see Supplemental Material, Table S3).

There was a significant correlation between the number of eosinophils per milligram of sputum and ECP (in nanograms per milliliter) (*p* < 0.001) (see Supplemental Material, Figure S3).

Compared with baseline measurements, ECP in sputum increased significantly after exposure to 600 ppb NO_2_ (43%; 95% CI: 17, 75%; *p* = 0.001) but not after exposure to clean air or 200 ppb NO_2_. However, the slope of the association between ECP and the level of exposure was not significant (Table 3).

Exposure to NO_2_ did not affect the other cell types (macrophages, neutrophils) measured in sputum (Table 3).

There was no correlation between methacholine challenge tests and eosinophil responses (data not shown).

## Discussion

Our findings indicate that repeated brief exposures to NO_2_ without allergen exposure increase eosinophilic airway inflammation in participants with intermittent asthma without inducing any changes in lung function. Because only mild asthmatics were tested, the results cannot be extrapolated to healthy individuals.

Although we cannot exclude that repeated challenges with hypertonic saline could have potentialized the effect of NO_2_, the repetition of sputum inductions is not the cause of the effect because the effect is expected to be the same for each of the three series of exposures (Pavord 1998). Eosinophils in sputum increased according to NO_2_ exposure level, and this significant trend supports a dose-related relationship. The effect on eosinophils and on ECP in sputum was significant at 600 ppb NO_2_. We found a strong correlation between ECP and eosinophils, suggesting that eosinophils were activated.

In participants with asthma, several studies have found that NO_2_ exposure increased eosinophilic inflammation in response to inhaled allergen, in the distal lower airways assessed by bronchial wash and bronchoalveolar lavage (BAL) (Barck et al. 2002), and in sputum (Barck et al. 2005). The three previous studies that have investigated effects of NO_2_ without allergen challenge in participants with asthma did not find changes in inflammatory cell distributions in BAL (Jorres et al. 1995) and in sputum (Solomon et al. 2004; Vagaggini et al. 1996). However, these studies involved small numbers of participants, and no repetition of exposure, and, in the study by Jorres et al. (1995), the evaluation of inflammation could have been performed too soon after exposure.

In the present study, participants with asthma were exposed to two realistic NO_2_ concentrations: 200 ppb and 600 ppb of NO_2_, which are close to NO_2_ peaks likely to be found indoors during the use of combustion appliances for cooking and heating (Basu and Samet 1999; Dennekamp et al. 2001; Kotzias et al. 2005; Pilotto et al. 1997) and outdoors for short periods in streets with heavy traffic and in road tunnels (Larsson et al. 2010). Exposures lasted 30 min, close to the average time spent cooking dinner in France (38 min during the week and 46 min the weekend in a 2003 survey) (Hébel 2012). Furthermore, exposure to intermittent peaks of NO_2_ may have greater effects than long-term, low-level exposure (Gardner et al. 1979).

Exposures in the present study were repeated over 2 days to mimic how exposures take place in real life and in order to assess a possible cumulative effect. At day 1, after one exposure, there was no significant change in eosinophilic airway inflammation—contrary to day 2 and day 3, after three exposures. These findings suggest that inflammatory response to NO_2_ exposure may be delayed or that a single exposure may be insufficient to enhance eosinophilic airway inflammation, suggesting a cumulative effect of NO_2_. These results are consistent with those of Barck et al. (2005) who found that two to three brief exposures at 260 ppb NO_2_ were needed to promote an increase in the airway inflammatory response to inhaled allergens. In addition to the study by Barck et al. (2005), our study shows that NO_2_, without an exposure to a high concentration of allergens, as with an allergen challenge, is sufficient to enhance inflammation in the airways. This finding could be of significance because exposure to peaks of NO_2_ is common, particularly indoors, whereas exposure to both high concentrations of NO_2_ and the specific stimulus for a susceptible individual is less likely (Hesterberg et al. 2009).

Many cities in Europe show an increase in concentrations of NO_2_ measured close to traffic due to the increasing number of vehicles, in particular diesel vehicles. Exhaust emissions from diesel vehicles are lower for carbon monoxide, non-methanic volatile organic compounds, and particulate matter but may be higher for NO_2_ (Guerreiro et al. 2012). Although epidemiological studies on NO_2_ have several limitations in particular because of the potential for exposure misclassification and co-pollutant effects, our results provide important evidence suggesting that NO_2_ alone has a direct effect on airway inflammation in asthmatics.

## Conclusions

To our knowledge, this is the first study to demonstrate that repeated peaks of NO_2_ at realistic concentrations without allergen exposure increase eosinophilic inflammation in the airways of asthmatics, supporting a dose–response relationship. Although it is difficult to evaluate the clinical implications of these findings in the present study, an increased eosinophilic inflammation may lead to exacerbation or loss of control of asthma (Green et al. 2002; Jacobsen et al. 2007). Therefore, we cannot rule out the effects of repeated exposures to NO_2_ over a longer period of time or effects in subpopulations.

## Supplemental Material

(271 KB) PDF
