# Supplementary material for: Repeated Nitrogen Dioxide Exposures and Eosinophilic Airway Inflammation in Asthmatics: A Randomized Crossover Study
Source: Environ Health Perspect. 2014 Apr 18;122(8):850–5. doi: 10.1289/ehp.1307240 (PMC4123022; doi:10.1289/ehp.1307240)
Supplement: (271 KB) PDF [file ehp.1307240.s001.pdf]

Environmental Health Perspectives doi:10.1289/ehp.1307240

Supplemental Material

Repeated Nitrogen Dioxide Exposures and Eosinophilic Airway Inflammation in Asthmatics: A Randomized Crossover Study

Véronique Ezratty, Gaëlle Guillossou, Catherine Neukirch, Monique Dehoux, Serge Koscielny, Marcel Bonay, Pierre-André Cabanes, Jonathan M. Samet, Patrick Mure, Luc Ropert, Sandra Tokarek, Jacques Lambrozo, and Michel Aubier

Table of Contents **Page**

Table S1. Sample sizes required to estimate statistically significant differences (alpha = 0.05) in percentage of eosinophils in sputum between two exposure groups with a variance of 0.10 and 80% power. **2**

Table S2. Absolute values not baseline adjusted (geometric means and 95% confidence intervals) for percentages of eosinophils measured in sputum (n = 16). **3**

Table S3. Changes from baseline [geometric mean percentage (95% confidence interval)], for the percentage of eosinophils in sputum with one participant excluded each time. **4**

Figure S1. Changes relative to baseline of eosinophil count and percentage of eosinophils for days 1, 2, and 3 (n=16). **5**

Figure S2. Change from baseline in the logarithm (base 10) of the percentage of eosinophils, for each participant (n=16). **6**

Figure S3. Correlation between eosinophil cationic protein (ECP) concentration in sputum supernatant (ng/ml) and the number of eosinophils per mg of sputum (n=16). **7**

Table S1. Sample sizes required to estimate statistically significant differences (alpha = 0.05) in percentage of eosinophils in sputum between two exposure groups with a variance of 0.10 and 80% power.

**Percentage increase in eosinophils**	**Sample size (n)**
50%	51
100% (doubling)	18
150%	10

Table S2. Absolute values not baseline adjusted (geometric means and 95% confidence intervals) for percentages of eosinophils measured in sputum (n = 16).

**Variable**	**0 ppb NO_2 (clean air)**	**200 ppb NO_2**	**600 ppb NO_2**	***p*-trend**
Percentage of eosinophils in sputum	3.92 (1.92, 8.01)	4.05 (1.99, 8.28)	6.44 (3.15, 13.2)	0.30

Table S3. Changes from baseline (geometric mean percentage (95% confidence interval)), for the percentage of eosinophils in sputum with one participant excluded each time.

Variable	0 ppb (clean air)	200 ppb	600 ppb	*p* trend
Participant 2 excluded	1% (-23, 32)	-5% (-27, 25)	77% (35, 132)	0.03*
Participant 3 excluded	-11% (-33, 18)	-4% (-28, 28)	63% (22, 117)	0.04*
Participant 4 excluded	-4% (-28, 27)	-1% (-26, 32)	71% (28, 129)	0.05*
Participant 5 excluded	-7% (-31, 24)	1% (-24, 35)	70% (27, 127)	0.05*
Participant 6 excluded	-13% (-35, 17)	-5% (-29, 27)	53% (14, 105)	0.06
Participant 7 excluded	-14% (-35, 14)	-5% (-28, 25)	48% (12, 97)	0.07
Participant 8 excluded	-20% (-40, 7)	-10% (-33, 2)	46% (8, 96)	0.05*
Participant 9 excluded	-15% (-35, 12)	-1% (-25, 3)	38% (4, 83)	0.09
Participant 11 excluded	-17% (-38, 11)	-3% (-27, 3)	52% (13, 104)	0.05*
Participant 12 excluded	-20% (-40, 7)	-13% (-35, 17)	53% (14, 106)	0.03*
Participant 13 excluded	-7% (-30, 24)	6% (-20, 41)	81% (35, 141)	0.03*
Participant 14 excluded	-22% (-42, 4)	-14% (-34, 15)	38% (3, 86)	0.06
Participant 15 excluded	-26% (-45, -1)	-19% (-39, 09)	40% (4, 89)	0.03*
Participant 16 excluded	4% (-21, 35)	25% (-5, 63)	62% (24, 113)	0.08
Participant 17 excluded	-13% (-45, 16)	-10% (-33, 21)	51% (13, 103)	0.06
Participant 19 excluded	-6% (-28, 24)	-12% (-33, 15)	71% (29, 125)	0.02*

Participants 1, 10 and 18 did not contribute and so were not included in the analysis.

*p-Value<0.05

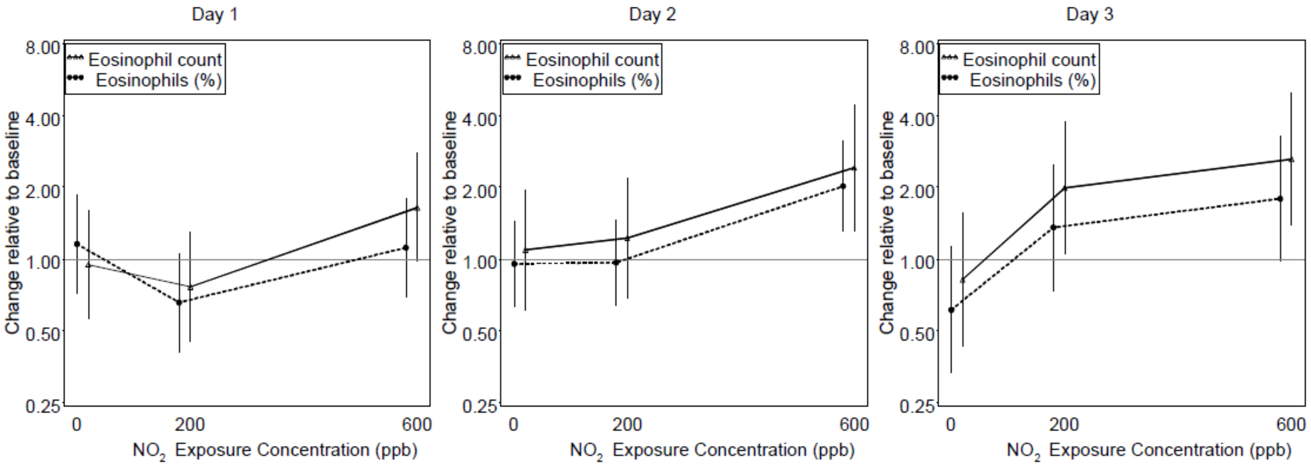

Figure S1. Changes relative to baseline of eosinophil count and percentage of eosinophils for days 1, 2, and 3 (n=16).

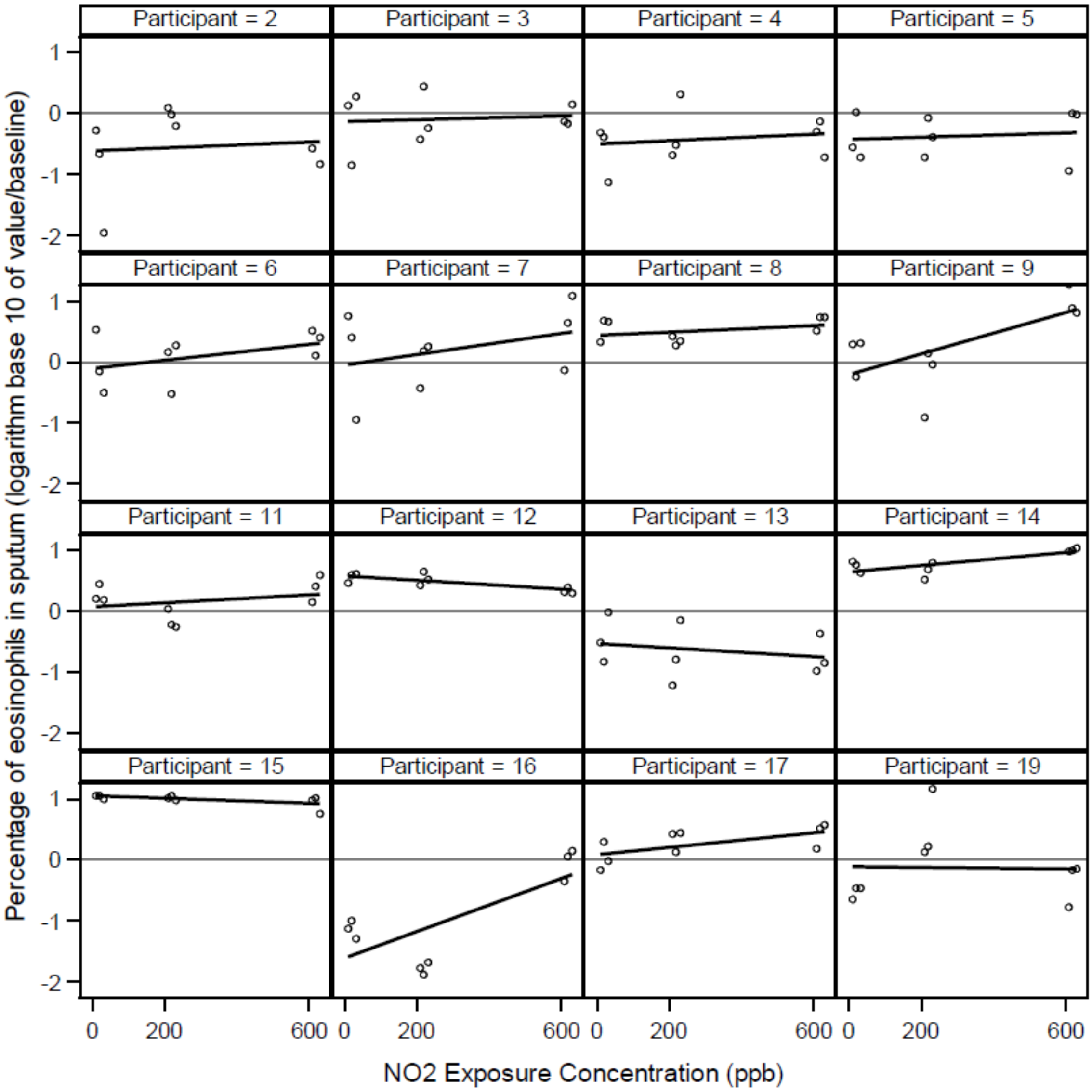

Figure S2. Change from baseline in the logarithm (base 10) of the percentage of eosinophils, for each participant (n=16). Individual plots are organized according to the subject number from subject 2 to subject 19. A change of 1 corresponds to a ten-fold increase from baseline. Each subject is identified by his or her percentage of eosinophils at baseline (measured 10 to 30 days before the first exposure).

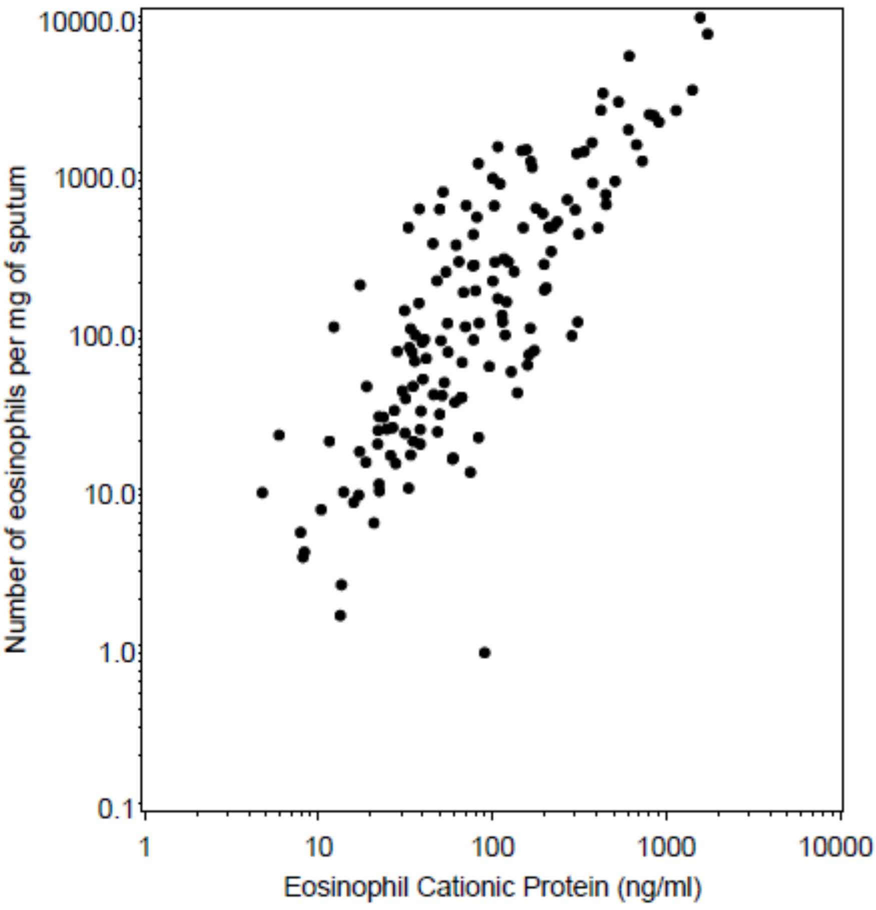

Figure S3. Correlation between eosinophil cationic protein (ECP) concentration in sputum supernatant (ng/ml) and the number of eosinophils per mg of sputum (n=16).
